# The impact of goal orientation on Chinese university students’ reading engagement: the mediating roles of boredom and self-efficacy

**DOI:** 10.3389/fpsyg.2026.1704593

**Published:** 2026-03-17

**Authors:** Li-Ching Hung, Xiaojie Lin, Meng-Te Hung, Cary Stacy Smith

**Affiliations:** 1 School of Cross-Border E-Commerce, Yango University, Fuzhou, China; 2 School of Liberal Arts, Minnan Normal University, Zhangzhou, China; 3 Department of Applied Psychology, Yango University, Fuzhou, China

**Keywords:** boredom, Chinese university students, goal orientation, reading engagement, self-efficacy

## Abstract

**Purpose:**

Drawing on achievement goal theory and self-efficacy theory, this study investigates how goal orientation shapes reading engagement, with a particular focus on the mediating roles of boredom and self-efficacy. While prior research has examined these factors separately, few studies have integrated them within a single analytical model, and even fewer have done so in non-Western higher education contexts.

**Methods:**

A cross-sectional survey was conducted with 522 undergraduates from universities in mainland China. Participants completed validated measures of goal orientation, boredom, self-efficacy, and reading engagement. Pearson correlations and path analysis were employed to test the hypothesized mediation model.

**Results:**

Goal orientation was positively associated with reading engagement (*β* = 0.42, *p* < 0.001). Boredom emerged as a significant negative mediator, whereas self-efficacy acted as a positive mediator. However, the dual mediation pathway of boredom and self-efficacy combined was not significant, indicating that motivational and affective processes may influence engagement through distinct channels.

**Conclusion:**

This study contributes new cross-cultural evidence on the mechanisms linking motivation, affect, and engagement in higher education. Findings underscore the importance of cultivating meaningful learning goals and strengthening students’ self-efficacy to sustain reading engagement, particularly in contexts where academic pressure is high. The study advances theoretical understanding by integrating motivational and emotional mediators within a unified model, offering insights relevant for both Chinese and international educational settings.

## 1 Introduction

Reading engagement is increasingly recognized as a central component of academic success and lifelong learning across diverse cultural contexts. In today’s information-saturated world, engagement with reading not only facilitates knowledge acquisition but also fosters critical thinking, adaptability, and personal development. International assessments such as PISA highlight that students’ levels of engagement in reading—encompassing interest, enjoyment, autonomy, and interaction—are strongly predictive of both immediate learning outcomes and long-term educational trajectories (Organisation for Economic Co-operation and Development, 2023, 2010).

Yet, growing international concerns have been raised regarding students’ reading engagement, particularly in higher education, where learners are required to process large volumes of complex texts. Emerging research suggests that sustained deep reading has declined in digitally mediated environments characterized by fragmented attention and multitasking (Delgado et al., 2018; Liu, 2005). These trends underscore the urgency of understanding the psychological mechanisms that promote or hinder reading engagement in contemporary university contexts.

Despite widespread acknowledgment of its importance, research on the mechanisms that sustain or hinder reading engagement has been uneven across contexts. Western studies have provided substantial insights into the role of motivational constructs such as goal orientation and self-efficacy in shaping reading behavior (Wigfield and Guthrie, 1997; Schunk and Zimmerman, 2007). However, relatively little attention has been devoted to how these mechanisms operate in non-Western cultural settings. Given that cultural norms—such as collectivism, hierarchical teacher–student relations, and high academic pressure—may significantly influence both motivation and affect, examining engagement in Asian higher education systems provides a valuable opportunity to extend existing theories.

In China, where education is positioned as a central driver of national development (Xue et al., 2021), university students are expected to demonstrate initiative, adaptability, and sustained engagement in learning (Han and Ye, 2017). Nevertheless, empirical evidence suggests that reading engagement among Chinese undergraduates is not uniformly high and may be influenced by contextual and emotional factors (Tang, 2015). As reading-intensive coursework remains foundational to university curricula, understanding the motivational and affective processes underlying reading engagement is both theoretically and practically significant.

Among the psychological factors influencing engagement, goal orientation has been identified as a critical construct for understanding how students set objectives and regulate effort (Pintrich, 2000). Complementing this, boredom—a pervasive but often neglected academic emotion—has been shown to undermine persistence and learning when tasks are perceived as monotonous or lacking value (Pekrun, 2006). Finally, self-efficacy, defined as individuals’ beliefs in their academic competence (Bandura, 1977), has been consistently linked to motivation, resilience, and engagement across domains.

Although prior research has examined these variables independently, studies have typically focused on partial relationships, such as the link between goal orientation and engagement (Pintrich, 2000), self-efficacy and academic persistence (Schunk and Zimmerman, 2007), or boredom within achievement emotion frameworks (Pekrun, 2006). However, few studies have systematically explored how goal orientation, boredom, and self-efficacy operate together within a unified explanatory model of reading engagement in higher education contexts. Existing research often isolates either motivational constructs (e.g., goal orientation and self-efficacy) or emotional factors (e.g., boredom), leaving the integrated pathways linking goals, affect, beliefs, and engagement underexplored. Moreover, such integrative models remain limited in non-Western higher education contexts.

To address this gap, the present study develops and empirically tests a mediation model examining the independent and combined roles of boredom and self-efficacy in linking goal orientation to reading engagement. By situating this analysis within Chinese higher education, the study contributes to the literature on reading engagement while extending achievement goal theory and self-efficacy theory across cultural contexts.

## 2 Literature review

### 2.1 The relationship between goal orientation and reading engagement

Goal orientation has long been recognized as a central motivational construct shaping learners’ persistence and engagement. Seminal scholars such as Dweck and Elliott (1983) and Pintrich (2000) argue that goal orientation reflects not only ability beliefs but also the broader cognitive and affective reasons individuals engage in achievement-related activities. More recent perspectives highlight that goal orientation influences learning behaviors through both motivational and emotional processes, extending beyond traditional ability-focused accounts (Pervin, 1983).

In reading contexts specifically, engagement has been conceptualized as a state of “flow,” characterized by immersion and intrinsic interest (Csikszentmihalyi, 1990). Consistent with this, Dweck and Leggett (1988) showed that mastery-oriented students demonstrate adaptability and sustained effort, while VandeWalle et al. (2001) reinforced its predictive value for proactive learning strategies and achievement outcomes. Yet, these insights have been derived largely from Western samples, with limited attention to domain-specific engagement such as reading. For example, O’Day and Rollefson (1995) linked learning motivation with academic quality broadly but did not differentiate between reading and other tasks.

Emerging evidence from non-Western contexts provides initial support for these relationships. For instance, Yang and Yang (2023) found that reading motivation promotes engagement and comprehension among Chinese students. Similarly, Jia et al. (2020) and Zhao et al. (2024) showed that strengthening goal orientation increases engagement. Recent empirical studies have further demonstrated that mastery-oriented goals are positively associated with domain-specific engagement and adaptive academic emotions in higher education settings (e.g., Liu and Zhou, 2024). However, these studies remain primarily descriptive and rarely investigate the underlying mechanisms. Moreover, affective experiences such as boredom and self-beliefs such as self-efficacy—both well-established in motivational theory—have not been systematically incorporated into models of reading engagement in Chinese higher education.

Taken together, while the existing literature confirms that goal orientation fosters engagement, important gaps remain. Most prior studies are rooted in Western contexts or treat engagement as a general learning behavior rather than a domain-specific phenomenon. Additionally, few studies consider how motivational and affective processes interact to explain sustained reading engagement in collectivist, high-pressure educational settings such as China. Addressing these gaps requires more integrative frameworks that examine both cognitive and emotional pathways linking goal orientation to engagement across cultural contexts.

### 2.2 The influence of boredom on the relationship between goal orientation and Reading engagement

Boredom has emerged as a complex affective state that can significantly undermine students’ learning engagement. Beyond being a fleeting mood, scholars have increasingly conceptualized boredom as reflecting unmet psychological needs and motivational deficits, consistent with self-determination theory (Deci and Ryan, 2000). From an existential perspective, it has also been described as a lack of purpose or meaning (Fahlman et al., 2009). Such perspectives converge on the idea that boredom is not merely passive disengagement but a signal of deeper misalignment between learners’ goals and their tasks. Boredom has been linked to reduced academic engagement and learning efficiency (Huang et al., 2011).

Early research suggested that boredom plays a mediating role in the relationship between motivation and academic behaviors. For example, Elliott and Dweck (1988) proposed that when tasks fail to align with students’ goals or provide sufficient challenge, boredom arises and disrupts engagement. More recent evidence supports this claim: achievement motivation has been found to negatively predict boredom (Ye et al., 2022). Recent empirical work further confirms that academic boredom functions as a significant mediator between motivational factors and engagement-related outcomes in higher education contexts (e.g., Liu and Zhou, 2024). Yet, the generalizability of this process across cultural contexts and specific academic domains remains underexplored.

From the perspective of control-value theory, achievement goals influence students’ perceptions of control and task value, which in turn shape achievement emotions such as boredom; these emotions subsequently affect engagement-related behaviors (Pekrun et al., 2010). When students hold clear and meaningful learning goals, they are more likely to perceive reading tasks as purposeful and valuable, thereby reducing boredom. Conversely, boredom, as a deactivating achievement emotion, can diminish attention, persistence, and effort investment, ultimately lowering reading engagement.

Luftenegger et al. (2016a,b) advanced this discussion by linking boredom to intrinsic motivation, goal orientations, and self-regulatory capacities. However, their results also highlighted variability across contexts, suggesting that boredom’s influence may not be uniform. In the Chinese context, empirical studies (Zhou and Chen, 2021; Liu, 2022) confirm that boredom diminishes engagement and learning efficiency, but these studies have been largely cross-sectional and have not explicitly examined boredom as a mediator within broader motivational frameworks.

Research outside China also points to similar patterns. For instance, Şi̇mşek et al. (2020) showed that boredom undermines concentration and academic performance, though their focus on mathematics leaves questions about domain-specific effects, such as in reading engagement. Recent theoretical developments in achievement emotion research also emphasize the dynamic and context-sensitive nature of boredom, suggesting that its impact may vary depending on task characteristics and cultural learning norms (Pekrun et al., 2010).

It is worth noting that boredom may also function as a moderator under certain theoretical frameworks. For example, modeled boredom as a moderator between self-efficacy and reading performance. However, the present study conceptualizes boredom as a mediator because our theoretical focus lies in explaining the motivational-to-emotional pathway through which goal orientation influences reading engagement. In this framework, boredom represents an emotional mechanism linking goals to behavioral investment rather than a boundary condition that alters the strength of existing relationships.

Taken together, the literature demonstrates a consensus that boredom negatively affects learning outcomes, but theoretical and empirical gaps remain. Specifically, few studies have systematically tested boredom as an intermediary between goal orientation and reading engagement, and even fewer have done so in collectivist, high-stakes educational settings.

Addressing this gap is essential, as boredom is not simply an individual emotional state but may be shaped by cultural, institutional, and pedagogical conditions. A more comprehensive framework that integrates both motivational and affective processes could illuminate how boredom operates as a barrier to sustained reading engagement across diverse cultural contexts.

### 2.3 The influence of self-efficacy on the relationship between goal orientation and Reading engagement

Self-efficacy, defined by Bandura (1977) as individuals’ beliefs in their capacity to successfully execute tasks, has been consistently recognized as a powerful predictor of learning engagement. Within social cognitive theory, self-efficacy is understood to shape the types of goals learners set, their resilience in the face of challenges, and the level of effort they are willing to sustain. In this way, self-efficacy functions as both a motivational driver and a behavioral regulator.

Prior research has shown that goal orientation and self-efficacy are closely intertwined. Elliot and McGregor (2001) argued that different goal orientations influence not only motivational tendencies but also self-beliefs about competence. Similarly, Linnenbrink and Pintrich (2003) observed that mastery-oriented students, who emphasize skill development and personal growth, tend to cultivate stronger self-efficacy compared to those who adopt performance-oriented goals. These findings underscore the importance of goal orientation as an antecedent to self-efficacy, but much of this work has been situated in Western contexts and focused on general learning outcomes rather than domain-specific engagement.

Evidence from related domains suggests the centrality of self-efficacy in sustaining academic effort. For example, Zimmerman and Bandura (1994) demonstrated that students with higher self-efficacy in writing displayed greater persistence and produced superior work, highlighting the role of confidence in creating a cycle of engagement and achievement. However, whether these dynamics extend to reading engagement—especially within collectivist educational contexts such as China—remains less clear.

Emerging studies provide encouraging evidence that self-efficacy is positively linked to engagement across contexts. Neff (2003) reported consistent positive associations between self-efficacy and student engagement. More recent empirical research has further demonstrated that self-efficacy significantly predicts domain-specific engagement in higher education settings (e.g., Liu and Zhou, 2024). Xie et al. (2021) showed that self-efficacy strengthens attitudes toward learning. In the Chinese higher education context, Liu and Zhou (2024) and Zhang et al. (2021) found a significant positive correlation between self-efficacy and learning engagement, and Cai and Jia (2020) demonstrated that self-efficacy mediates the relationship between goal orientation and learning engagement. These findings suggest that self-efficacy may represent a critical mechanism through which motivational orientations translate into sustained engagement.

Nevertheless, gaps remain. Few studies have systematically examined self-efficacy in the context of reading engagement, despite reading being a core academic activity in higher education. Moreover, while prior research often treats self-efficacy and boredom as separate constructs, little empirical work has considered their potential joint or interactive roles in mediating the link between goal orientation and engagement. Recent scholarship continues to support the centrality of self-efficacy in sustaining engagement across academic domains (Pekrun et al., 2017; Wang et al., 2024). Addressing these gaps is crucial, particularly in cultural settings where both high academic pressure and collectivist norms may shape how self-beliefs operate in relation to motivation and emotion.

### 2.4 The mediating effects of boredom and self-efficacy on the relationship between goal orientation and Reading engagement

Although prior studies have consistently demonstrated that both boredom and self-efficacy serve as mediators between goal orientation and academic outcomes, far less is known about how these factors may operate in tandem, particularly in the context of reading engagement. Luftenegger et al. (2016a,b) emphasized that boredom diminishes engagement by dampening intrinsic motivation and interest, while Haycock et al. (1998) highlighted that self-efficacy enhances academic performance through persistence and resilience. However, these findings have been largely limited to Western populations and to domains such as mathematics or writing, leaving the applicability to reading in non-Western contexts underexplored. Recent scholarship has increasingly called for integrative models that simultaneously account for motivational beliefs and achievement emotions in explaining engagement processes (Pekrun et al., 2017; Putwain et al., 2018). Nevertheless, empirical studies that incorporate both boredom and self-efficacy within a single mediation framework remain limited, particularly in domain-specific reading contexts.

A further unresolved issue is whether boredom and self-efficacy act as independent mediators or interact within a combined process. Some research has suggested that boredom stems from unmet autonomy needs and low perceived competence (Deci and Ryan, 2000), positioning it as a negative affective state that undermines goal-directed behavior. Empirical findings further indicate that boredom is negatively associated with engagement and persistence across subject areas (Tze et al., 2016). In contrast, Bandura (1997) conceptualized self-efficacy as a personal resource that strengthens motivation and buffers against negative emotions, potentially offsetting the disengaging effects of boredom. These divergent perspectives underscore the need for a more integrative approach that considers whether these constructs work independently or interactively in shaping reading engagement.

In sum, although both boredom and self-efficacy have been identified as important mediators of academic outcomes, their joint effects in the specific domain of reading engagement remain unclear. Investigating this relationship is theoretically significant because it advances understanding of how motivational and affective processes intersect to influence sustained academic involvement. It is also practically important for educators seeking strategies to enhance engagement by simultaneously reducing negative affect and fostering positive self-beliefs. Situated in the Chinese university context, the present study addresses this gap by examining whether boredom and self-efficacy operate as parallel or combined mediators between goal orientation and reading engagement. While prior Western research has established robust links between goal orientation and engagement (e.g., Schunk and Zimmerman, 2007), relatively little is known about whether these mechanisms generalize across collectivist educational cultures. Clarifying this issue contributes not only to cross-cultural theory-building but also to the design of interventions tailored to diverse sociocultural learning environments.

### 2.5 Research hypotheses and model

#### 2.5.1 Research hypotheses

Based on the preceding literature, the following hypotheses were proposed:

*H1*: Goal orientation is positively associated with reading engagement.

*H2*: Boredom mediates the relationship between goal orientation and reading engagement.

*H3*: Self-efficacy mediates the relationship between goal orientation and reading engagement.

*H4*: Boredom and self-efficacy jointly mediate the relationship between goal orientation and reading engagement.

#### 2.5.2 Research model

See Figure 1.

**Figure 1:**
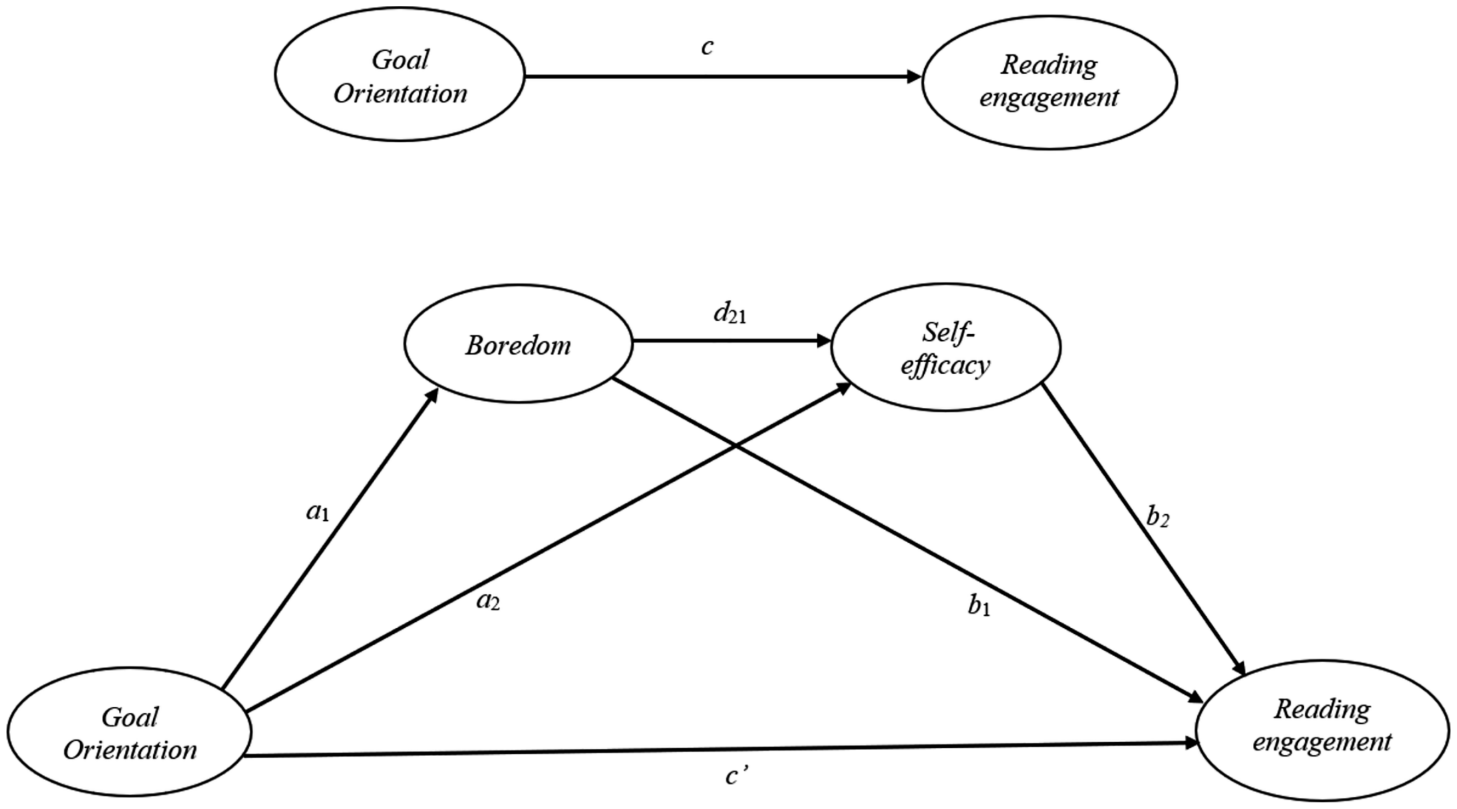
The statistical model in which the effect of goal orientation on reading engagement is mediated by boredom and self-efficacy.

## 3 Methods

### 3.1 Participants

Data were collected using the Wenjuanxing online survey platform, which distributed questionnaires nationwide across mainland China. A total of 522 valid responses were obtained from university students representing diverse institutions, majors, and academic levels. Among the participants, 397 were female and 125 were male, with ages ranging from 18 to 29 years (*M* = 20.38, *SD* = 1.73). The majority were Han Chinese (*n* = 490), while 32 students reported belonging to ethnic minority groups. In terms of academic standing, 141 were first-year students, 186 sophomores, 90 juniors, 74 seniors, 26 postgraduate students, and 5 at other levels. Disciplinary backgrounds were also varied: 146 majored in science, engineering, agriculture, or medicine; 250 in literature, history, philosophy, or education; 85 in economics, management, or law; and 41 in other fields. This diversity in demographics, academic disciplines, and institutions increases the representativeness of the sample within the Chinese university context, although generalization beyond China should be made with caution.

### 3.2 Procedure

Participants were invited through online distribution links on Wenjuanxing. Before beginning the survey, they were provided with an informed consent statement explaining the purpose of the study, the voluntary nature of participation, and the guarantee of anonymity and confidentiality. Only those who provided consent proceeded to complete the questionnaire. All items were presented in Chinese, and validated scales with established reliability were used. On average, the survey required approximately 15 min to complete. Responses were automatically recorded and screened for missing data and inattentive response patterns. Ethical approval for the study was obtained from the first author’s university research ethics committee.

### 3.3 Measures

#### 3.3.1 Self-efficacy scale

**Figure 2:**
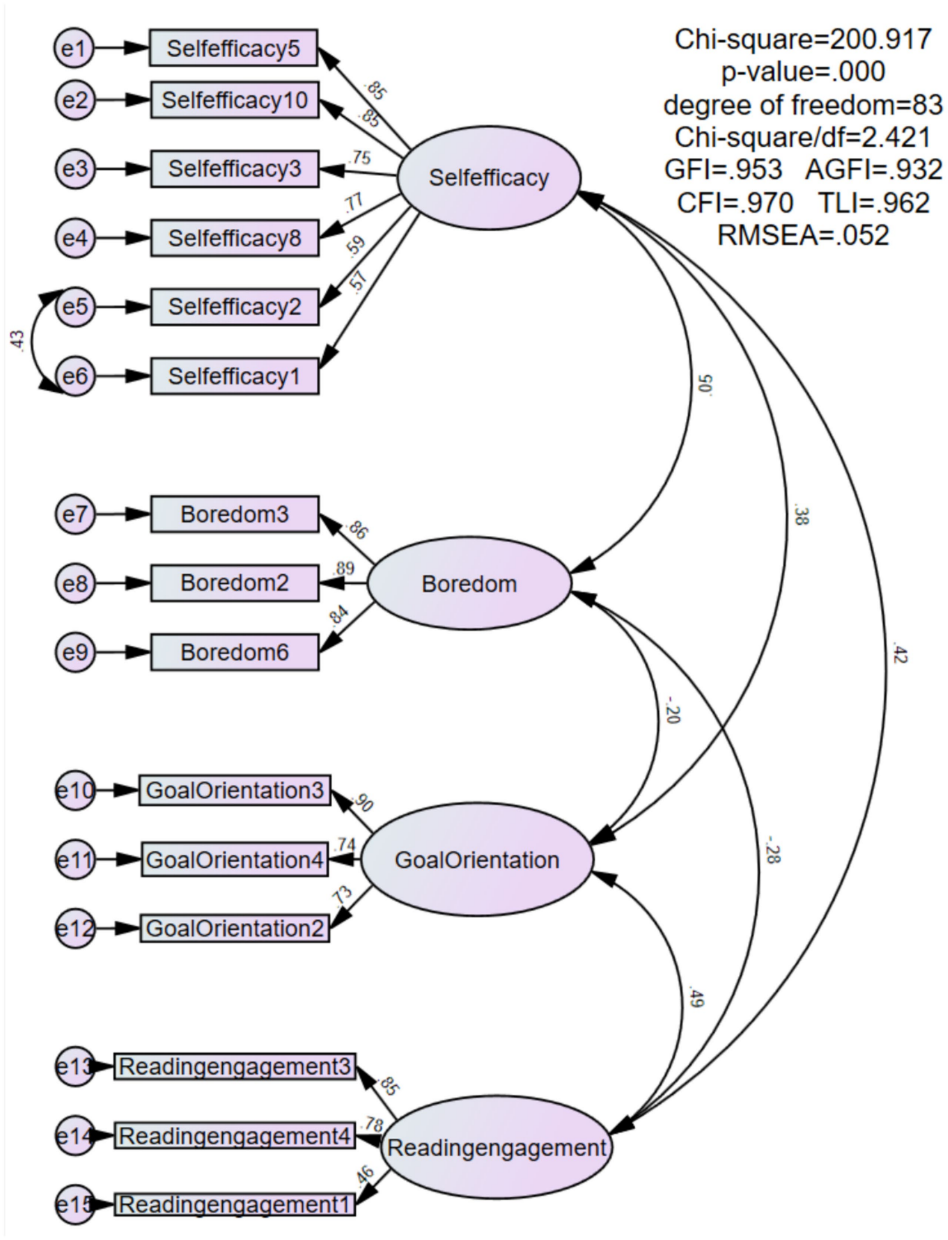
Confirmatory factor analysis: CFA measurement model (*N* = 522).

The Self-Efficacy Scale used in this study adopts the Chinese version translated by Zhang and Schwarzer (1995). The original scale was created by Jerusalem and Schwarzer in 1981 with 20 items and was reduced to 10 items in 1992 (Jerusalem and Schwarzer, 1992). The scale contained 10 items scored on a 7-point Likert scale (1 = *Strongly Disagree* to 7 = *Strongly Agree*). Previous research has demonstrated strong internal consistency (Cronbach’s alpha = 0.82; Chong et al., 2018). Although Bandura’s (1977, 1997) theoretical framework emphasizes domain-specific self-efficacy, generalized self-efficacy reflects individuals’ overall confidence in coping with academic challenges and has been widely applied in educational research. In the present study, self-efficacy was conceptualized as a generalized academic competence belief that may influence students’ persistence and engagement in reading-related tasks. Therefore, the use of the General Self-Efficacy Scale is theoretically aligned with the broader conceptualization of efficacy beliefs guiding motivated behavior. To ensure reliability and validity in this study, confirmatory factor analysis (CFA) was conducted (Hair et al., 2010). Four items with poor performance were removed, resulting in a final version of six items (see Figure 2). Reliability analysis indicated that the scale demonstrated good internal consistency (Cronbach’s alpha = 0.823; see Table 1).

**Table 1:** Summary of the reliability and validity analysis for study variables (*N* = 522).

Measure	*α*	CR	AVE	Pearson correlations			
				1	2	3	4
1. Self-efficacy	0.823	0.876	0.546	**0.739**			
2. Boredom	0.881	0.896	0.743	0.050	**0.862**		
3. Goal orientation	0.725	0.834	0.628	0.382***	−0.201***	**0.793**	
4. Reading engagement	0.725	0.752	0.518	0.416***	−0.281***	0.493***	**0.720**

***p < 0.001; **p < 0.01; *p < 0.05. α = Cronbach’s alpha (α) coefficient. CR, composite reliability; AVE, average variance extracted. The square roots of the AVEs appear in bold on the diagonal. Correlations are shown below the diagonal.

#### 3.3.2 Boredom scale

Boredom was measured using items adapted from the Chinese version of Academic Emotion Questionnaire developed by Pekrun et al. (2002). The scale has previously shown excellent reliability (Cronbach’s alpha = 0.92). Responses were collected on a 7-point Likert scale (1 = *Strongly Disagree* to 7 = *Strongly Agree*). Following CFA, three poorly performing items were excluded, and the final scale included three items (see Figure 2). The Cronbach’s alpha coefficient for this study was 0.881, indicating high reliability (see Table 1).

#### 3.3.3 Goal orientation scale

Goal orientation was assessed using the Goal Orientation subscale from the Chinese version of the Motivated Strategies for Learning Questionnaire developed by Pintrich et al. (1991), which was translated by Tong and Kember (2019). After adaptations, the scale consisted of four items and demonstrated adequate reliability in previous studies (Cronbach’s alpha = 0.67; Jackson, 2018). The items were rated on a 7-point Likert scale (1 = *Strongly Disagree* to 7 = *Strongly Agree*). CFA was performed, resulting in the removal of one poorly performing item and a final version with three items (see Figure 2). The Cronbach’s alpha coefficient in this study was 0.725, demonstrating moderate reliability (see Table 1).

#### 3.3.4 Reading engagement scale

Reading engagement was measured using items based on the Chinese version of the student questionnaire responses from PISA 2018 (Organisation for Economic Co-operation and Development, 2019). Responses were rated on a 5-point Likert scale (1 = *Strongly Disagree* to 5 = *Strongly Agree*), with higher scores indicating greater reading engagement. Previous research has reported acceptable internal consistency (Cronbach’s alpha = 0.66; Gong, 2021). In this study, CFA led to the exclusion of three items with inadequate performance, resulting in a final scale of three items (see Figure 2). The Cronbach’s alpha coefficient was 0.725, indicating moderate reliability (see Table 1).

### 3.4 Data analysis

This study employed a comprehensive set of descriptive statistical analyses to provide an overview of participants’demographic characteristics. Pearson correlation analyses were conducted to examine the relationships among the key variables, including goal orientation, boredom, self-efficacy, and reading engagement.

To further investigate how goal orientation indirectly influences university students’ reading engagement, we developed and rigorously tested a mediation model specifying three distinct pathways. The PROCESS macro for SPSS was used to estimate the mediating effects, ensuring the scientific rigor and precision of the analyses.

In addition, to assess the model’s fit and explanatory power, multiple fit indices were applied. These included the significance of the chi-square (*χ^2^*) statistic, the proximity of the Tucker-Lewis Index (TLI) and Comparative Fit Index (CFI) to recommended thresholds, and the minimal values of the Standardized Root Mean Square Residual (SRMR) and Root Mean Square Error of Approximation (RMSEA). These stringent evaluation criteria jointly provided a robust basis for assessing the quality of the proposed model.

Overall, by employing a multi-level and multi-dimensional analytical strategy, this study comprehensively examined the complex associations between goal orientation and reading engagement and tested the hypothesized mediation mechanisms.

## 4 Results

### 4.1 Model fit analysis

A comprehensive measurement model was constructed to evaluate the four variables—goal orientation, boredom, self-efficacy, and reading engagement—and the model’s fit to the sample data was thoroughly assessed. The fit indices indicated that the proposed model achieved an acceptable level of fit: *χ*^2^/df = 2.421, RMSEA = 0.052, SRMR = 0.052, CFI = 0.970, and PClose = 0.333 (Hu and Bentler, 1999). Detailed values for all fit indices are presented in Table 2.

**Table 2:** Model fit measures (*N* = 522).

Measure	Estimate	Threshold	Interpretation
CMIN/DF	2.421	Between 1 and 3	Excellent
CFI	0.970	>0.95	Excellent
SRMR	0.052	<0.08	Excellent
RMSEA	0.052	<0.06	Excellent
PClose	0.333	>0.05	Excellent

CMIN, chi-square; DF, degree of freedom; CFI, comparative fit index; SRMR, standardized root mean square residual; RMSEA, root mean square error of approximation.

Additionally, all standardized factor loadings were statistically significant, ranging from 0.46 to 0.90 (*p* < 0.001). Most factor loadings exceeded the threshold of 0.5 recommended for evaluating measurement models in academic research (Hair et al., 2010).

These results suggest that the measurement model provided an acceptable fit to the data, meeting commonly accepted standards for model evaluation in empirical studies and providing an adequate methodological foundation for subsequent analyses. However, consistent with prior methodological guidance (Hair et al., 2010), model fit indices and statistical significance may be influenced by sample size; therefore, the results should be interpreted with appropriate caution.

### 4.2 Construct validity

Following established research protocols, the construct validity of the instruments was assessed using both convergent and discriminant validity criteria. For convergent validity, three core indicators were evaluated: item loadings, composite reliability (CR), and average variance extracted (AVE). As shown in Figure 2, the factor loadings for all items ranged from 0.46 to 0.90, confirming strong item reliability and ensuring the accuracy of the measurement results (Hair et al., 2010).

Furthermore, the computed CR values for the latent constructs ranged from 0.752 to 0.896, exceeding the widely accepted threshold of 0.70 and indicating high internal consistency and structural stability of the scales. The AVE values ranged between 0.518 and 0.743, surpassing the recommended minimum of 0.50 (Hair et al., 2010), thereby confirming that the constructs met the standards for convergent validity.

For discriminant validity, the study adhered to the guidelines proposed by Fornell and Larcker (1981). Specifically, the square roots of the AVE values for each latent variable were confirmed to be greater than their correlations with other latent variables. As presented in Table 1, all AVE square roots exceeded the corresponding inter-construct correlation coefficients, clearly demonstrating the distinctiveness and independence of each construct. These findings provided strong evidence of the study’s excellent discriminant validity.

### 4.3 Correlation, path and mediation analysis

As shown in Table 3, goal orientation was positively correlated with reading engagement (*r* = 0.422, *p* < 0.001) and self-efficacy (*r* = 0.364, *p* < 0.001). Importantly, boredom was negatively correlated with both goal orientation (*r* = −0.179, *p* < 0.001) and reading engagement (*r* = −0.234, *p* < 0.001). These negative correlations indicate that students with higher levels of goal orientation tend to experience lower levels of boredom during reading activities. Likewise, higher boredom is associated with lower reading engagement, suggesting that boredom functions as a disengaging emotional state in academic contexts. However, boredom was not significantly correlated with self-efficacy (*r* = 0.022, *p* > 0.05).

**Table 3:** Descriptive statistics and Pearson correlation matrix results for all variables (*N* = 522).

Measures	*M*	*SD*	1	2	3	4
1. Reading engagement	2.932	0.763	1			
2. Goal orientation	5.169	1.129	0.422***	1		
3. Self-efficacy	4.689	1.027	0.341***	0.364***	1	
4. Boredom	3.764	1.436	−0.234***	−0.179***	0.022	1

***p < 0.001.

**Figure 3:**
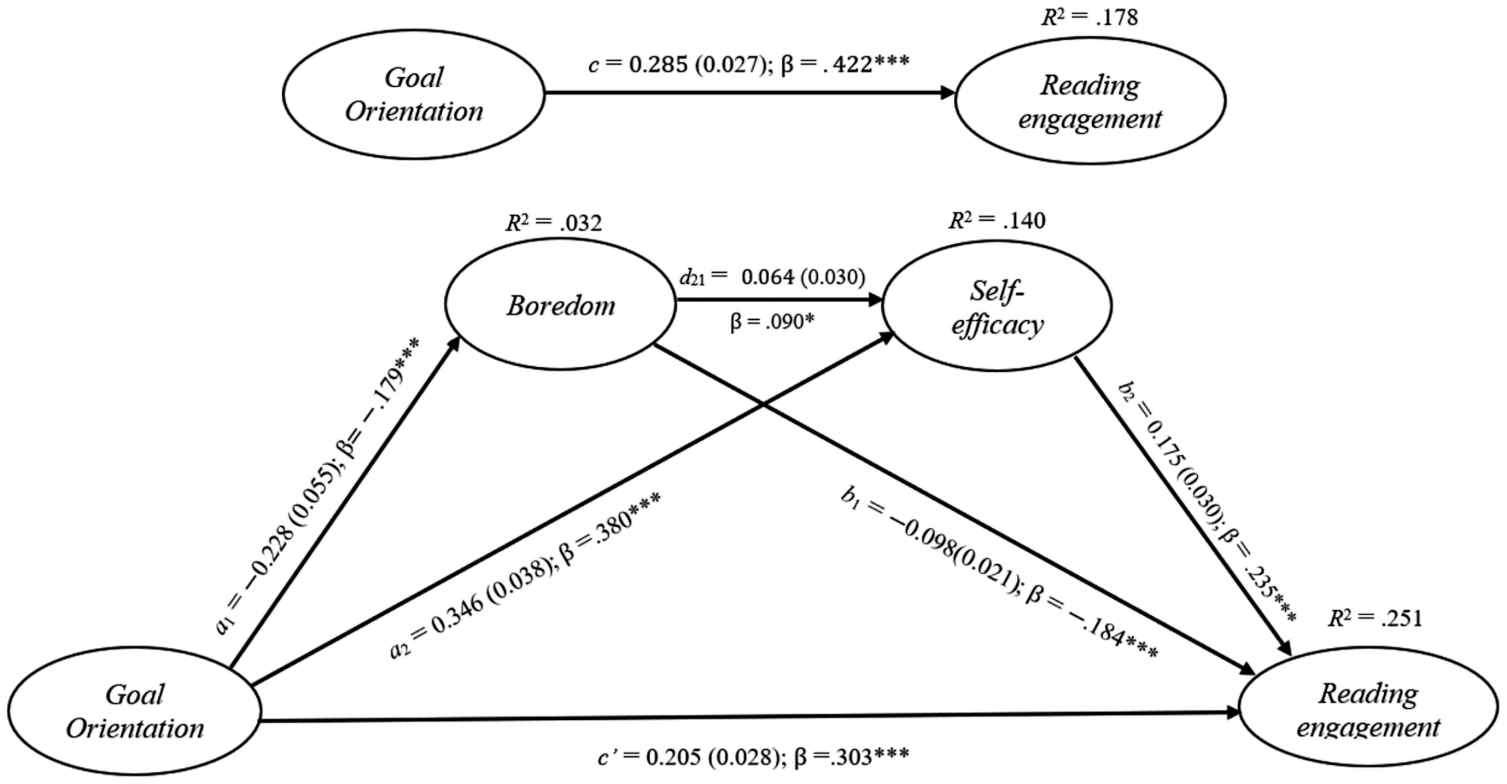
Model summary information for the Hypothesized Mediation Model Portrayed in Figure 1 (*N* = 522). Standardized coefficients (*β*) and unstandardized regression coefficients *a_1_*, *a_2_*, *b_1_*, *b_2_*, *c*, *c’*, and *d_21_* are presented along with their standard errors (shown in parentheses). *p < 0.05. **p < 0.01 ***p < 0.001.

The results of path analysis indicated that goal orientation had a significant predictive effect on college students’ reading engagement, with *c* = 0.285, *SE* = 0.055, *β* = 0.422, *p* < 0.001, and *R^2^* = 0.178 (as shown in Figure 3 and Table 4). These findings provide empirical support for H1. When the two variables of boredom and self-efficacy were introduced, although the standardized regression coefficient of goal orientation on self-efficacy decreased, it still maintained a significant predictive effect, with *c* = 0.205, *SE* = 0.028, *β* = 0.303, *p* < 0.001, and *R^2^* = 0.251 (as shown in Figure 3 and Table 4).

**Table 4:** Summary of the research mediation model (*N* = 522).

Outcome									
Predictor	Boredom (Mediator 1)			Self-efficacy (Mediator 2)			Reading engagement		
	*B* (*β*)	*SE*	*p*	*B* (*β*)	*SE*	*p*	*B* (*β*)	*SE*	*p*
Goal orientation	*a_1_*→−0.228 (−0.179)	0.055	<0.001	*a_2_*→0.346 (0.380)	0.038	<0.001	*c*’→0.205 (0.303)	0.028	<0.001
Boredom				*d_21_*→0.064 (0.090)	0.030	0.030	*b_1_*→−0.098 (−0.184)	0.021	<0.001
Self-efficacy							*b_2_*→0.175 (0.235)	0.030	<0.001
Constant	4.944	0.290	<0.001	2.660	0.245	<0.001	1.423	0.188	<0.001
	*R*^2^ = 0.032 (1, 520) = 17.292 *p* < 0.001			*R*^2^ = 0.140 *F*(2, 519) = 42.270 *p* < 0.001			*R*^2^ = 0.251 *F*(3, 518) = 57.880 *p* < 0.001		

B, Unstandardized regression coefficient; *β*, Standardized regression coefficient.

In testing the mediating effect model, this study adopted the bootstrap method in the PROCESS macro for SPSS software package, which is highly recognized in the field of statistics. In the specific implementation process, 10,000 repeated samplings were performed to estimate the stability and significance of the indirect effect. Table 5 presents the statistical results of the mediating effect, specifying the effect size, standard error, and 95% confidence interval of each path.

**Table 5:** Summary of the mediation model analysis (*N* = 522).

Effect type	Mediator	Equation	Point estimate	95% bootstrap CI		
				*SE*	*LL*	*UL*
Indirect effect 1	Boredom	*a*_1_ × *b*_1_	0.022	0.009	0.007	0.044
Indirect effect 2	Self-efficacy	*a*_2_ × *b*_2_	0.060	0.016	0.033	0.093
Indirect effect 3	Boredom + Self-efficacy	*a*_1_ × *d*_21_ × *b*_2_	−0.003	0.002	−0.006	0.000
Total indirect effect		*a*_1_*b*_1_ + *a*_2_*b*_2_ + *a*_1_*d*_21_*b*_2_	0.080	0.016	0.052	0.114

Effect type	Equation	Point estimate	95% CI		
			*SE*	*LL*	*UL*
Direct effect	*c’*	0.205	0.028	0.150	0.260
Total effect	*c* = *c’* + *a*_1_*b*_1_ + *a*_2_*b*_2_ + *a*_1_*d*_21_*b*_2_	0.285	0.027	0.232	0.338

CI, confidence interval; LL, lower limit; UL, upper limit. If the confidence interval does not include zero, it reveals a significant effect.

The inhibitory effect of goal orientation on boredom was very significant (a_1_ = −0.228, *SE* = 0.055, *β* = −0.179, *p* < 0.001, *R^2^* = 0.032), and boredom also had a significant effect on reading engagement (*b_1_* = −0.098, *SE* = 0.021, *β* = −0.184, *p* < 0.001).

According to the data in Table 5, in the study of the effect of goal orientation on reading engagement, with boredom as the mediating variable, the mediating effect was *a_1_* × *b_1_* = 0.022, and the 95% bootstrap CI was [0.007, 0.044]. Since the 95% bootstrap CI did not contain 0, the mediating effect was established (as shown in Table 5). These results provide evidence supporting H2.

Meanwhile, goal orientation also had a significant impact on self-efficacy (*a_2_* = 0.346, *SE* = 0.038, β = 0.380, *p* < 0.001), and self-efficacy had a significant promoting effect on reading engagement (*b_2_* = 0.175, *SE* = 0.031, β = 0.235, *p* < 0.001). With self-efficacy as the mediating variable, the mediating effect was *a_2_ × b_2_* = 0.060, and the 95% bootstrap CI was [0.033, 0.093]. Since the 95% bootstrap CI did not contain 0, the mediating effect reached a significant level, that is, it had a mediating effect (as shown in Table 5). These findings further support H3.

In addition, boredom could also predict self-efficacy (*d_21_* = 0.064, *SE* = 0.030, β = 0.090, *p* < 0.05). When boredom and self-efficacy were taken as chain mediating variables, according to the data in Table 5, the mediating effect was *a_1_* × *d_21_* × *b_2_* = 0.003, and the 95% bootstrap CI was [0.006, 0.000]. Since the 95% bootstrap CI contained 0, the mediating effect was not significant, and H4 was not empirically supported by the present data.

In addition to statistical significance, the coefficients of determination (*R*^2^) provide important information regarding the explanatory power of the model. The *R*^2^ value for reading engagement was 0.251, indicating that 25.1% of the variance in reading engagement was explained by goal orientation, boredom, and self-efficacy. In social and educational research, where behavior is shaped by multiple psychological and contextual influences, *R*^2^ values in the range of 0.10 to 0.30 are generally considered to reflect moderate explanatory power. Therefore, although the *R*^2^ values may appear modest, they represent meaningful levels of variance explained in the context of complex human learning processes.

In summary, goal orientation significantly predicts college students’ reading engagement. Boredom and self-efficacy function as independent mediators between goal orientation and reading engagement; however, the proposed chain mediation pathway was not empirically supported.

## 5 Discussion and conclusion

### 5.1 Discussion

This study examined the relationships among goal orientation, boredom, self-efficacy, and reading engagement in Chinese university students. By integrating motivational and affective constructs within a single analytical model, it addressed a notable gap in the literature, as few prior studies have simultaneously tested these mediating mechanisms, particularly in non-Western contexts.

Consistent with earlier findings (Ames and Archer, 1988; Miller et al., 1993), the results confirmed that goal orientation positively predicts reading engagement. Students who establish clear learning goals are more likely to persist and invest sustained effort. Importantly, these results extend existing knowledge by showing that such positive associations are robust in the Chinese higher education context, thereby contributing new cross-cultural evidence to achievement goal theory. Recent research has similarly emphasized the role of mastery-oriented goals in fostering domain-specific engagement across cultural contexts (Putwain et al., 2018), further supporting the robustness of this relationship.

The mediating role of boredom aligns with Elliott and Dweck’s (1988) proposal that disengagement arises when goals lack meaning or sufficient challenge. The present findings add nuance by situating boredom specifically within the reading domain, highlighting that monotonous or irrelevant tasks can undermine engagement even among otherwise motivated students. This interpretation is consistent with recent meta-analytic evidence indicating that boredom is negatively associated with academic engagement and achievement (Tze et al., 2016). This suggests that educators should design reading activities that are meaningful, challenging, and contextually relevant to sustain motivation.

The role of self-efficacy corroborates Bandura’s (1997) social cognitive theory and subsequent work (Linnenbrink and Pintrich, 2003; Zimmerman and Bandura, 1994), which emphasize that self-beliefs foster persistence and achievement. By confirming self-efficacy as a key mediator, this study reinforces its importance in shaping reading engagement and demonstrates that motivational processes remain central even in academically high-pressure contexts such as China. Recent scholarship continues to support the centrality of self-efficacy in sustaining engagement across academic domains (Pekrun et al., 2017).

Interestingly, boredom and self-efficacy did not jointly mediate the relationship between goal orientation and reading engagement. This diverges from some motivational models (e.g., Luftenegger et al., 2016a, 2016b) that posit synergistic effects of affect and cognition. A possible explanation is that in the Chinese context, boredom reflects situational disengagement while self-efficacy represents more stable competence beliefs, leading them to operate along distinct pathways. Another interpretation is cultural: collectivist values emphasizing diligence and persistence may buffer against the compounding effects of boredom and low self-efficacy. Future cross-cultural research is needed to test whether these independent pathways generalize across contexts or whether cultural norms moderate the relationships observed here.

Overall, this study makes several theoretical contributions. First, it extends achievement goal theory by showing that motivational (self-efficacy) and affective (boredom) processes can independently link goals to engagement, rather than working synergistically. Second, it provides empirical support for self-determination theory (Deci and Ryan, 2000), suggesting that both the fulfillment of competence needs (via self-efficacy) and the prevention of negative affect (via boredom) are critical for sustained engagement. This integrative perspective aligns with contemporary calls for models that simultaneously account for cognitive and emotional mediators in academic engagement (Pekrun et al., 2017). Third, by situating the analysis in Chinese higher education, the study advances cross-cultural educational psychology, demonstrating both the universality and cultural specificity of motivational and emotional mechanisms.

Taken together, these findings encourage researchers and practitioners to address both cognitive and affective dimensions of learning engagement, and they highlight the importance of testing psychological models across diverse cultural and institutional contexts.

### 5.2 Theoretical implications

This study contributes to theory in several ways. First, it extends achievement goal theory by demonstrating that goal orientation continues to serve as a robust predictor of learning engagement in the domain of reading, a relatively underexplored area compared to mathematics and writing. Importantly, this finding in a Chinese higher education context underscores the cross-cultural applicability of the theory and provides evidence that motivational orientations are influential even in collectivist cultures that emphasize external expectations and conformity.

Second, the results refine self-efficacy theory by showing that self-efficacy operates as a key mediator between goal orientation and reading engagement. This supports Bandura’s (1997) proposition that self-beliefs drive persistence and resilience but also highlights the relevance of self-efficacy in specific academic domains such as reading.

Third, by incorporating boredom as an affective mediator, the study expands existing motivational models. Whereas prior research has often emphasized the interaction between cognitive and affective factors, the present findings suggest that boredom and self-efficacy may exert distinct and parallel influences. This nuance enriches theoretical debates about whether motivational and affective processes combine synergistically or operate independently in predicting engagement.

Finally, the cross-cultural focus of this study enhances the ecological validity of motivational and affective frameworks. By situating the analysis in the Chinese higher education system, the findings reveal how cultural norms—such as perseverance under academic pressure—may shape the pathways linking motivation, emotion, and engagement. This cross-cultural evidence contributes to building more inclusive theories of student engagement that move beyond Western-centric assumptions.

### 5.3 Practical implications

The findings of this study offer several implications for educators, policymakers, and families seeking to enhance student engagement in reading, both within China and in other educational contexts.

First, the significant role of goal orientation suggests that instructional practices should emphasize the development of clear and meaningful learning goals. Universities can support this by embedding structured goal-setting workshops, reflective learning activities, and formative assessment practices into their curricula. Teachers who provide ongoing feedback and opportunities for self-reflection can help students connect personal goals to broader academic outcomes, thereby strengthening purpose and persistence.

Second, boredom emerged as a salient barrier to engagement, underscoring the need for instructional innovation. Educators should diversify reading tasks by integrating interactive formats such as collaborative projects, multimedia resources, and problem-based learning. Universities can also leverage digital platforms that allow students to select texts aligned with both personal interests and curricular demands, thereby reducing monotony and promoting autonomy.

Third, the mediating role of self-efficacy highlights the importance of interventions that build students’ confidence in their reading abilities. Scaffolded instruction, opportunities for incremental mastery, and constructive feedback are especially valuable in cultivating positive self-appraisals. Faculty development programs can equip instructors with strategies such as attribution retraining, which encourages students to link success to effort and strategy rather than innate ability.

Fourth, families contribute to engagement by fostering a supportive home reading culture. Parents can encourage positive attitudes toward reading through shared reading activities, open discussions about texts, and consistent routines that normalize reading as part of daily life. Setting realistic but challenging expectations also reinforces motivation and self-belief.

Finally, at the policy level, initiatives that promote a culture of reading engagement are crucial. This could include funding national reading campaigns, developing regional reading hubs, and incentivizing higher education institutions to design innovative reading programs. By situating reading engagement as both an academic and societal priority, such initiatives can contribute to the cultivation of lifelong learners who are better equipped to thrive in rapidly changing knowledge economies.

Taken together, these implications suggest that reading engagement is best promoted through a multi-level approach that integrates motivational, affective, and contextual supports. Importantly, while the study focused on Chinese university students, the strategies outlined here can inform educational reforms and interventions across diverse cultural and institutional settings.

### 5.4 Limitations and future research

Despite its contributions, this study has several limitations that should be acknowledged and addressed in future research.

First, the sample consisted exclusively of Chinese university students. While this focus provides valuable insights into engagement dynamics in a collectivist, high-pressure educational context, it also limits the generalizability of the findings. Future research should replicate this model in diverse cultural and educational settings to examine whether the observed relationships hold across contexts. Comparative studies involving Western and non-Western samples would be particularly useful for clarifying how cultural norms, such as collectivism and academic competition, shape motivational and affective processes.

Second, the use of a cross-sectional survey design precludes definitive conclusions about causality. Although path analysis provided evidence of potential mediation, longitudinal or experimental designs are needed to capture how goal orientation, boredom, and self-efficacy interact over time. Future research could, for example, track students across semesters or test interventions aimed at enhancing self-efficacy to evaluate causal pathways more robustly.

Third, this study concentrated on individual-level psychological factors. Yet, engagement is also shaped by broader social and institutional contexts. Future work could adopt multi-level approaches to examine how classroom climate, peer norms, or institutional policies interact with individual traits to influence reading engagement. Such models would extend understanding from a purely psychological perspective to a more ecological one.

Fourth, although this study was theoretically grounded in Bandura’s conceptualization of self-efficacy, we employed a generalized self-efficacy measure rather than a domain-specific reading self-efficacy scale. While generalized efficacy beliefs capture overall confidence in coping with academic challenges, domain-specific measures may provide more precise insights into reading-related competence beliefs. Future research could incorporate task-specific or reading-specific self-efficacy instruments to further refine the explanatory power of the model.

Finally, boredom and self-efficacy were modeled here as parallel mediators, but more complex dynamics may exist. For instance, boredom could gradually erode self-efficacy, or personality traits might moderate the strength of these pathways. Exploring moderated mediation or longitudinal growth models could provide richer insights into the interplay between motivation and affect in academic engagement.

By addressing these limitations, future studies can build on the present findings to develop a more nuanced and culturally sensitive understanding of reading engagement. Such efforts would not only refine theoretical frameworks but also inform interventions aimed at fostering sustained motivation and well-being in diverse educational contexts.

### 5.5 Conclusion

This study investigated how goal orientation influences reading engagement among Chinese university students, with particular attention to the mediating roles of boredom and self-efficacy. The results demonstrate that goal orientation significantly predicts engagement and that boredom and self-efficacy independently mediate this relationship. These findings contribute to the literature by integrating motivational and affective factors into a single model and by extending research on reading engagement to a non-Western educational context.

Theoretically, the study highlights that boredom and self-efficacy may function through distinct pathways rather than synergistically, pointing to the need for more nuanced models of student engagement. Practically, the results suggest that interventions should simultaneously address affective challenges, such as reducing boredom, and cognitive resources, such as strengthening self-efficacy. These strategies may be relevant not only for educators and policymakers in China but also for those in other contexts seeking to promote sustained engagement in reading and other learning domains.

More broadly, this research underscores the importance of examining how motivational and emotional mechanisms operate across diverse cultural settings. By clarifying the processes linking goal orientation, boredom, and self-efficacy to engagement, the study contributes to cross-cultural educational psychology and provides a foundation for comparative work across different societies. Future research that extends these insights to varied cultural, institutional, and disciplinary contexts will be critical for building more globally informed models of student engagement and academic development.

## Data Availability

The datasets presented in this study can be found in online repositories. The names of the repository/repositories and accession number(s) can be found at: https://doi.org/10.5281/zenodo.16613834.
